# Hepatocellular carcinoma: from hepatocyte to liver cancer stem cell

**DOI:** 10.3389/fphys.2015.00154

**Published:** 2015-05-18

**Authors:** Ioannis Karakasiliotis, Penelope Mavromara

**Affiliations:** ^1^Molecular Virology, Hellenic Pasteur InstituteAthens, Greece; ^2^Molecular Biology and Genetics, Democritus University of ThraceAlexandroupolis, Greece

**Keywords:** HBV, HCV, hepatocellular carcinoma, liver, stem cells

Self-renewal and the potential to yield various cell types are the main attributes of stem cells and shared by the so called cancer stem cells (CSCs). CSCs consist a small portion of cells within the tumor mass that are very often resistant to chemotherapy and radiation, while being responsible for cancer relapse after treatment. The role of cancer stem cells has been recognized in cancers such as leukemia and colon cancer, whereas their role in hepatocellular carcinoma (HCC) remains to be unraveled. The vast majority of HCCs (~80%) are related to Hepatitis virus B (HBV) and C (HCV) persistent infection implicating parallel pathways of intrinsic cell transformation and chronic infection-inflammation.

CSCs reside among other more differentiated cancer cells, are resistant to apoptosis and their self-renewal is based on transcription factors similar to those of adult and embryonic stem cells such as Oct4, Nanog and Klf-4. Although their origin and relationship with the tumor initiating cell is still debatable it is rather established that they sustain tumor growth, produce differentiated progenies that organize a hierarchical cell system and induce eventually tumor's metastatic potential. Recently, it has been suggested that persistent oncogenic viruses such as HBV and HCV trigger the formation of CSCs that are possibly responsible for the high rates of recurrence and mortality that characterize HCC.

Both HBV and HCV although very different in their genome organization and life cycle they result often in persistent infection that leads eventually in 1–5% of infected individuals to HCC through liver fibrosis and cirrhosis. HBV replicates its DNA in cell nucleus and remains episomally in the form of minichromosome. HBV DNA fragments are often integrated in host genome. HCV on the other hand is a positive strand RNA virus with cytoplasmic replication organized in virus induced vesicles. HBx protein of HBV and HCV core, NS3, and NS5A proteins are modulators of cellular pathways that lead to cell transformation. HBV and HCV chronic infection induce accumulative liver damage followed by an increasing liver regeneration that is favored by both viruses. Indeed, hepatic progenitor cells increase their numbers in the liver of HCV patients as the disease advances to cirrhosis (Tsamandas et al., 2006), while liver CD133+ (a stemness surface marker) CSCs correlated with early recurrence and poor prognosis among HBV related HCC patients (Ma et al., 2010). During cirrhosis, HBV and HCV proteins support the survival and sustain the growth of infected cells within a mayhem of inflammatory-mediated cell death.

Replication of HCV in Huh7.5 hepatoma cells in the form of a subgenomic replicon, which bears only the non-structural proteins of HCV NS3-5B, showed marked induction of liver stem cell markers such as Lgr5, cytokeratin-19 (CK-19) doublecortin and CaM kinase-like-1 (DCAMKL-1) and CD133 in conjunction with liver progenitor cell makers such as α-fetoprotein (AFP). Remarkably, curing the cells by using Interferon 2a reversed the phenomenon, while xenograft-derived tumors of these cells in mice encompassed cells with liver progenitor and CSC traits (Ali et al., 2011). Biopsies from HCV-linked tumors supported the presence of DAMKL-1+ cells, whose abundance increased with the disease stage (Ali et al., 2011). Concurrently, expression of HBx protein of HBV in HepG2 hepatoma cells greatly enhanced the abundance of the pluripotency transcription factors such as Oct-4, Nanog, and Klf-4, as well as the “stemness”-associated markers EpCAM and β-catenin (Arzumanyan et al., 2011). Biopsies from HBV infected patients recapitulated the overexpression of EpCAM, while Oct-4 and Nanog expressing cells were detected within the tumors (Arzumanyan et al., 2011). Recently, Nanog was shown to be induced by HCV core protein through activation of STAT3. HCV core facilitated cell cycle progression in a Nanog-dependent manner (Zhou et al., 2014). Interestingly, Nanog was upregulated in the liver of NS5A expressing mice, while the effect was maximized during alcohol feeding in a TLR4-dependent manner (Machida et al., 2009).

The induction of stemness by HCV structural and non-structural proteins through parallel pathways possibly underscores its importance in virus proliferation and persistence. Huh7.5 cells support HCV replication, in contrast to their predecessors Huh7, to a great extent due to the constitutive activation of sonic hedgehog pathway (Choi et al., 2011), which is known to be critical for liver regeneration and the control of CSC self-renewal. Although one cannot exclude the contribution of innate immunity and mir-122 in cell permissiveness to HCV. A more direct effect of stemness-related factors in HCV replication was observed by siRNA knockdown of DCAMKL-1 which correlated well with the decline in HCV RNA abundance (Ali et al., 2011).

Conditions within the microinviroment of the infected cells could also play an important role in the programming of liver cells toward CSCs and the divergence of the multiple pathways induced by HCV and HBV toward epithelial to mesenchymal transition (EMT) or stemness. Such condition, which is often present in inflamed and malignant areas of the liver, is hypoxia. Accumulated evidence from various cancer types strongly support the hypothesis that hypoxia sustains the self-renewal characteristics of a portion of cancer cells in hypoxic niches mainly due to the upregulation of Oct4, NANOG, SOX2, Klf4, and c-myc (Mathieu et al., 2011; Muz et al., 2014). HBV and HCV modulate hypoxic pathways to adapt cells in hypoxic conditions conferring EMT characteristics (Wilson et al., 2011; Kim, 2014). Remarkably, hypoxic conditions induced HCV replication in Huh7.5 cells which correlated with activation of anaerobic cell energy production and cell proliferation (Vassilaki et al., 2013). Thus, interplay between the induction of stemness conditions through hypoxia and activation of cell energy status by HCV and through that enhancement of HCV replication may lead to a boost of both HCV spread and tumorigenesis in chronically infected individuals.

A less evaluated parameter in HCV and HBV HCC are the extrinsic signals of CSC sustainment. Cytokines such as IL-8 are secreted during HBV infection, while they fluctuate during disease exacerbation periods (Dunn et al., 2007). HCV NS5A could also induce IL-8 production and elevated IL-8 levels were linked to resistance in interferon therapy (Polyak et al., 2001a,b). IL-8 is known to sustain CSC self-renewal in cancers such as breast and pancreatic cancer increasing invasiveness (Singh et al., 2013; Chen et al., 2014). Tumor associated macrophages (TAMs) are possibly part of the niche that sustains CSCs in breast cancer and HCC (Fan et al., 2014; Lu et al., 2014). Especially for HCC, TAMs sustain CSCs (EpCAM+ cells) through TGFb1 induction of EMT (Fan et al., 2014). HBx and HCV core are the key proteins in the induction of TGFb1 signaling-dependent EMT markers, while HCV core diverts TGFb1 signaling from tumor suppression to EMT induction (Battaglia et al., 2009). IL-6 is secreted by inflammatory and stromal cells during liver regeneration and it is known to support the conversion of non-CSCs to CSCs (Kim et al., 2013). In breast cancer IL-6 promoted self-renewal and hypoxia survival through activation of Notch pathway (Sansone et al., 2007). Both HCV and HBV induce IL-6 production, while increased IL-6 levels are poor prognostic marker for either treatment outcome (HCV, Guzman-Fulgencio et al., 2012) or HCC severity (HBV, Chang et al., 2015). The link between IL-6 and stemness was furthermore supported by the high positive correlation between IL-6 levels and Oct4/Nanog expression in HBV HCC patients (Chang et al., 2015). IL-6 sustainment of HCC progenitor cell phenotype seems to apply even in chemically induced HCC as stemness-associated markers and cell proliferation are linked to autocrine IL-6 activation (He et al., 2013).

Thus, it is becoming increasingly supported that HBV and HCV infection promotes EMT and stem cell-like characteristics in the infected liver cells that enable them to adapt and proliferate in the hypoxic inflammatory environment of the cirrhotic liver paving the ground for the occurrence of HCC (Figure 1). These characteristics seem to favor the presence of the virus in the infected liver and are possibly associated with persistent viral replication. As recurrence and mortality are still hallmarks of HCC, it is becoming a central question to clarify the network of the intrinsic induction of CSC phenotype through the activation of EMT and stemness transcription factors and the extrinsic sustainment of this phenotype by inflammatory and regenerative signals. Identification of those characteristics that differentiate liver CSCs from normal adult stem cells and liver progenitors and dissection of the CSC niche microenvironment are critical for the development of new therapies that target the root of HCC and its potential to reinitiate tumor formation after treatment.

**Figure 1:**
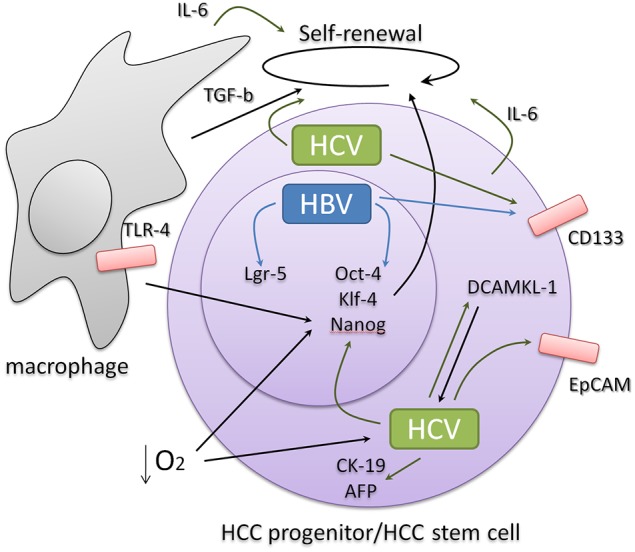
**HBV and HCV infection induce the expression of stemness and progenitor-related cell markers derived either from intrinsic or extrinsic signals that involve liver inflammation and regeneration**. Signals related to pluripotency often feedback favoring viral abundance.

## Conflict of interest statement

The authors declare that the research was conducted in the absence of any commercial or financial relationships that could be construed as a potential conflict of interest.
